# Does attention bias modification reduce anxiety in socially anxious college students? An experimental study of potential moderators and considerations for implementation

**DOI:** 10.1371/journal.pone.0264256

**Published:** 2022-02-25

**Authors:** Klavdia Neophytou, Georgia Panayiotou

**Affiliations:** Department of Psychology, University of Cyprus, Nicosia, Cyprus; Julius-Maximilians-Universität Würzburg, GERMANY

## Abstract

According to cognitive models, preferential attention to social threat contributes to maintenance of social anxiety. Socially anxious individuals are known to show attention biases to threatening stimuli, although there is inconsistency in the literature with regards to the type of attentional biases they present. This study examines the effect of attention bias modification (ABM) for social anxiety in non-treatment-seeking college students meeting social anxiety disorder criteria, taking into consideration previous mixed results regarding its effectiveness. Attention bias levels and types (i.e. vigilance vs avoidance) at baseline were examined and considered as potential moderators of ABM effects. Sixty-eight socially anxious individuals were randomly allocated to ABM vs placebo groups. A structured interview and self-report assessment were completed at pre-treatment and post-treatment. Results showed half of the participants presented few attention biases at baseline, and the rest presented either vigilance or avoidance. Participants with low attention biases scored higher in social anxiety than those showing avoidance and there was no difference between those showing vigilance vs avoidance. No significant effects from pre to post treatment were observed in attention biases, self-report or structured interview of anxiety in the ABM group. Baseline attention biases did not moderate these effects. Results are discussed with regards to implications for future research towards the creation of more effective protocols, based on the needs of heterogeneous social anxiety sub-groups.

## Introduction

Social anxiety disorder (SAD) is highly prevalent with rates estimated around 5% of the general population [1]. It is considered as the second most common psychological disorder in the general US [2] and European [3] population, and has been related to economic costs, work absenteeism and high health care utilization [4]. According to DSM-5, individuals with SAD can be divided into those demonstrating primarily performance or public speaking fears and those with general social fears, who are the majority of cases [5,6]. Individuals with either sub-type have similar age of onset, family history and sociodemographic characteristics [2]. SAD is typically chronic and disabling [7], a risk factor for the development of additional psychopathology, and shares high comorbidity with other anxiety disorders and depression [8,9].

Cognitive-behavioral therapy is the most effective treatment for adults with social anxiety [10]. However, a high percentage of them, that could reach over 80%, do not seek or receive treatment [1,11]. In addition to other documented reasons, such as distance from psychological services, or unwillingness to share personal information with strangers, individuals with SAD avoid treatment because of fears of negative evaluation from the therapist [12]. These considerations create the need for novel treatment approaches that may be more widely acceptable, either as stand alone, or as complements to cognitive-behavioral therapy [13].

Attention bias modification (ABM) is a promising intervention which could potentially help overcome these obstacles. This is a computerized intervention, eliminating concerns about face-to-face interactions, at least during early stages of treatment when anxiety is highest. It is suggested to be more easily accessible, as it requires no or limited therapist involvement and can be completed quickly. ABM aims to reduce anxiety by changing attentional processes: It trains anxious individuals to focus their attention on emotionally neutral or pleasant stimuli, based on the assumption that attention biases towards threatening stimuli play a role in the etiology and maintenance of anxiety disorders [14].

This assumption is supported by different theoretical models [15,16], which have been used to support the idea that ABM can be a useful stand-alone component in the overall treatment of SAD, or an auxiliary to highly effective cognitive-behavioral interventions. The Cognitive-Behavioral model of anxiety [16] notes that audience feedback e.g. a perceived angry face that socially anxious individuals pay preferential attention to, reinforces the negative image they hold of how they may look to others, creating more anxiety. Therefore, allocating less attention to such cues, and increased attention to neutral or positive audience feedback may be crucial in changing negative self-images. At the same time, attentional bias is believed to moderate the impact of negative life events on stress and anxiety [17,18]. Anxious individuals appear to allocate preferential attention to even milder threat conditions compared to non-anxious individuals, and this may prolong and perpetuate stress, making the world appear less safe, with ramifications for health and wellbeing. For these reasons, further investment on procedures that may modify these biases is worthwhile, as a means to complement existing effective treatments.

In the case of social anxiety, ABM trains individuals to focus attention less on angry or other threatening faces [13,19] and more on neutral or happy faces instead. However, compared to the large number of studies supporting the effectiveness of ABM for other anxiety disorders and trait anxiety [20–22], a smaller number of studies found supportive evidence when it comes to social anxiety, [e.g. 13,19,23–27], with some studies finding non-significant changes from pre to post-treatment on attention biases and SAD symptoms, suggesting no or little effectiveness [28–32; see metanalysis in 33]. It is possible that reasons for these mixed results pertain to the nature of attention biases associated with social anxiety.

Although the association between anxiety and attentional biases towards threat have been frequently reported in the past [34], more recent evidence casts doubt on their robustness, [35], at least when it comes to the dot-probe task. Results are somewhat more mixed when it comes to social anxiety, with some studies finding no evidence of attention biases [36,37], but several studies showing the expected pattern of greater attention towards social threatening information [38]. However, in addition to the typical hyper-vigilance to threat found in other anxiety conditions, studies find additional attention bias patterns in SAD, including difficulty in disengaging from threat, and threat avoidance, suggesting heterogeneity of bias in this anxiety disorder [36,39]. According to more recent theorizing, this heterogeneity, but also the absence of more consistent attention bias findings, may reflect individual differences that remain stable across situations, or a dynamic shift in attention biases within the same individual, as the emotional situation evolves [40,41]. To some degree, reliability issues of the way attention bias is calculated within the dominantly used dot-probe task, may account for these inconsistencies [40,41]. According to a recent review [37], additional moderators of the type of attention bias shown by socially anxious individuals include the type and timeframe of stimulus presentation, as well as social anxiety level.

More specifically, in addition to hyper-vigilance toward socially threatening information, including signs of disapproval from others [13,39], socially anxious individuals also have difficulties with disengaging their attention from threat [42]. This process refers to a delayed withdrawal of attention from a stimulus because of its ability to hold attention [43]. In fact, it was suggested that this may be the only attentional bias relevant to SAD [44]. In addition, there is evidence that socially anxious individuals show attentional avoidance of threatening stimuli, a pattern that is more uniquely related to SAD than other anxiety disorders, [37], and it is dominant in several theoretical models of SAD [15,45–48].

Given these considerations, it is useful to further examine the role of heterogeneity in attention biases in social anxiety, in the mixed results regarding the effectiveness of ABM. Focusing to start with on heterogeneity at the level of individuals, it is possible, that traditional ABM works only for the subset of socially anxious participants with strong attentional vigilance [29,49]. Indeed, there is evidence that participants with limited vigilance toward threat do not show substantial gains [50]. These individual differences in the degree attentional bias towards threat, as well as the existence of other types of attention bias as part of the psychopathology of SAD, on which ABM may be less effective, have been largely ignored in most studies [see 50 and 51 for exceptions] creating the need for additional research.

In addition to the above, the mixed findings regarding the effectiveness of ABM for SAD may be attributed to methodological differences among studies. Because of the promising results for other anxiety conditions, the majority of ABM studies recruited treatment-seeking participants, and presented ABM as an effective new treatment [e.g. 24,26], which may have created demand effects on outcomes. Strong expectancy effects may overshadow specific treatment effects, leading to similar reduction in reported anxiety, in both control and intervention group. Only one study [24] showing significant effectiveness of ABM recruited non-treatment seeking participants (college students with high levels of social anxiety). It appears that more experimental studies on non-treatment seeking samples are required to establish the effects of ABM on various components of attention biases, before this intervention can be presented as an effective part of treatments for SAD.

This study aims to examine the effects of ABM, in comparison to a placebo condition, on social anxiety symptoms and pre-intervention heterogeneity in attention biases, in non-treatment seeking students with high levels of social anxiety. The widely used dot-probe task was used to assess attention biases and deliver ABM [18,52]. Because of the possibility that differences in levels of attention biases towards, vs avoidance of social threat, shown by each individual may moderate effectiveness, attention bias levels, assessed at baseline, were examined. Specifically, baseline level of vigilance vs avoidance was assessed, using widely available calculation methods [e.g., 14,52,53] for purposes of comparability to the studies showing mixed effectiveness results. In order to reduce expectancy effects of presenting this as an intervention for SAD, any terminology including ‘intervention’, ‘therapy’ etc. was avoided. Instead, participants were told that they would participate in an experiment to see if an attentional task aiming to change attentional processes can produce changes in their anxiety levels. Further examining the effectiveness of ABM in well-controlled experimental studies and addressing the role of baseline attention bias can have important implications for clinical research, towards the design of more effective ABM protocols that are specific to the mechanisms sustaining anxiety among socially anxious individuals.

Taking extant research into consideration, it was expected that: 1) heterogeneity at the individual level could be observed in attention biases at baseline, so that some participants would show more vigilance, and others would show more avoidance of threat; different levels of social anxiety may be associated with different degrees of vigilance vs avoidance, although prior hypotheses cannot be set due to the mixed findings, 2) the ABM group, in comparison to the placebo control group at post-treatment would present: a) reduction in vigilance to threat as assessed by reaction time (RT) to the dot-probe, b) reduction in SAD symptoms, as measured by structured interview and self-report, 3) baseline levels of attention bias would moderate ABM effects, with decreased attention to threat and reduced SAD symptoms [self-report and interview] found to a greater degree among participants with more vigilance to threat at baseline, compared to those with greater avoidance.

## Materials and method

### Participants

The sample was comprised of undergraduate students who took part in the experiment for extra credit or participation in a lottery for a tablet. Participants who scored above the clinical cut-off of 28 on the Difference subscale of the Social Phobia and Anxiety Inventory–23 (SPAI-23), or scored one standard deviation above the mean on the social anxiety subscale (M = 21, SD = 12) [54] and agreed to participate in the study, were interviewed using the Anxiety Disorders Interview Schedule adult version (ADIS-IV) [54] to confirm that their anxiety was in the clinical range. Only those who met ADIS-IV SAD criteria were included, to ensure high levels of symptoms. Exclusion criteria were the presence of current: 1) suicidal intent, 2) substance abuse, 3) meeting diagnostic criteria for post-traumatic stress disorder, obsessive-compulsive disorder, 4) past schizophrenia, bipolar disorder, organic mental disorder, and 5) any concurrent psychotherapy, 6) changes in medication during the 12 weeks prior to study, and 7) CBT during the 6 months before the study. The first four criteria were assessed with ADIS-IV and the last three criteria were assessed through a short interview developed for this study.

In total 68 participants were selected from the screening sample for their high SPAI-23 scores. Eight of them were removed for not meeting ADIS-IV criteria for SAD. Finally, the ABM group consisted of 32 participants and the placebo group of 28 participants (total N = 60), to which they were double-blindly assigned. Participants met criteria for SAD (20 participants) or the SAD specifier in DSM-5, i.e. performance anxiety (40 participants) on the basis of ADIS-IV.

### Procedure

The screening study was announced in classes, along with an invitation to the experimental phase. Additional students were recruited during mental health screening days of the University Mental Health Center, where students filled out the SPAI-23 as part of screening and were informed about the study. Students meeting SPAI-23 criteria from either sample were invited to the study and those who consented were interviewed as above. Interviewers in all cases were trained, doctoral level clinical psychology students. At the end of the data collection, every interview was checked between interviewers and the primary researcher to confirm that inclusion and exclusion criteria were met. Informed consent was obtained for all study stages, which were approved by the National Bioethics Committee.

Upon arrival to the lab, participants were informed that they would perform an attention task. They were also told that the study would assess if the task can result in changes of their attentional processes (where they focus their attention) depending on their group allocation, and that it would assess if these changes in attention focus can result in changes of their anxiety levels, which they knew were considered high relative to the general population from the discussion of their ADIS-IV results. At the end of the experiment, participants were asked about their assumptions with regards to the training that they received, in order to assess their blindness to the ABM vs placebo condition. Most of them (90%) selected the ‘do not know’ option, suggesting blindness to group assignment.

To complete the study, participants came to the lab for 8 sessions: The first session (lasting approximately 1.5 hours) included: interview with the ADIS-IV; participants who did not meet clinical levels (8 participants) were provided an explanation with regards to the study’s criteria and were dismissed. Those meeting inclusion criteria completed the baseline attention bias assessment through the dot-probe task. Next, they completed a package of questionnaires assessing their baseline anxiety levels (described below). Lastly, they received either the active ABM (training away from threat) or placebo training depending on their group assignment.

Over the next 3-weeks, participants received the ABM or placebo training, depending on their group, a total of 6 times, twice a week, preceded by computerized instructions. Sessions lasted about 7 minutes. During the last (8^th^) session, participants received again the ABM or placebo training as usual; next they completed the self-report questionnaires (post-intervention assessment) and their attentional biases were re-assessed using the dot-probe task similar to baseline. Lastly, participants were re-assessed with the ADIS-IV. The last session lasted approximately 1.5 hours.

### Measures

A battery of self-reported and clinician-rated measures of anxiety and dysfunction were the dependent variables at pre and post intervention.

*Anxiety Disorders Interview Schedule adult version* (ADIS-IV) [55], is a structured diagnostic interview assessing anxiety disorders according to DSM–IV. It also assesses/screens for depression, somatoform disorders and substance abuse, psychotic symptoms and family psychiatric history. This interview shows excellent reliability for social anxiety [56]. It was administered to confirm that participants had significant (clinical) levels of social anxiety and to rule out other diagnoses according to the study’s exclusion criteria.

*Social Phobia and Anxiety Inventory-23* (SPAI-23) [57], is a shortened version of the SPAI [58] measuring symptoms of social anxiety. It consists of two sub-scales: social phobia (16 items) and agoraphobia (7 items). Each item is rated on a 5-point scale from 0 (never) to 4 (always). The Agoraphobia sub-scale can be subtracted from the social phobia scale, producing a Difference score. A Difference score ≥28 is indicative of clinical levels of SAD. The two scales have good internal consistency and adequate test re-test reliability over 5 1/2 weeks (r = 0.72; social phobia sub-scale, α = .95; agoraphobia sub-scale, α = .85) [57,58], and good internal consistency in Greek-Cypriot adolescents (social phobia sub-scale, α = .95; agoraphobia sub-scale, α = .85) [59]. For the current study, subscale internal consistency was as follows. Pre-treatment: social phobia, α = .90; agoraphobia, α = .85. Post-treatment: social phobia, α = .93; agoraphobia, α = .86).

*Liebowitz Social Anxiety Scale Test* (LSAS) [60] is a 24 item self-report measure rated on a 4-point scale. It assesses fear and avoidance in social and performance situations during the past week. It yields an overall total score and six sub-scale scores: total fear, fear of social interactions, fear of performance situations, total avoidance, avoidance of social interaction, avoidance of performance situations. In the current study, only the total score was used. Scores of 55–65 indicate moderate, 65–80 marked, 80–95 severe social anxiety and score greater than 95 is indicative of marked social phobia. The LSAS has overall good psychometric properties (test–retest reliability, convergent and discriminant validity), with internal consistency α = 0.95, and is sensitive to treatment change [61]. It also showed very good internal consistency, and replicated factor structure in a Greek-Cypriot student sample collected by the researcher (unpublished data; Cronbach’s α = 0.94). For this study, pre-treatment α = 0.95, post-treatment α = 0.96.

### The dot probe attention task

The dot-probe task was adopted from the Tel Aviv University/ National Institute of Mental Health (TAU/NIMH) study. Specifically, the task includes face photographs of 20 individuals (10 male, 10 female) taken from the NimStim stimulus set [62], with the exception of one female picture taken from the Matsumoto and Ekman set [63]. Emotionally valent (anger) and neutral faces were selected. Threatening facial expressions are considered an ancient signal for submissiveness in evolutionary history [64], and are therefore fearful, especially to individuals with social anxiety. Angry faces are often used in studies of SAD [for such studies using the TAU/NIMH protocol see 23,25,65]. During the task, pairs of stimuli, angry-neutral faces or neutral-neutral faces of the same actor are presented vertically in the centre of the screen. Two different sets of pictures (A or B) are used, one for assessment and the other during training, counterbalanced between subjects. Faces are presented against a plain black background as in the Matsumoto and Ekman set and each picture is 45mm width x 34mm height. Each face is distanced from its pair by14mm. Photographs are centered vertically, with equal distance to the top and bottom of the fixation cross. The top face is 20mm from the top edge of screen. Photographs are surrounded by a single 58mm wide x 94mm tall white rectangle, which shows the area of the screen that the participant needs to focus on. Stimulus presentation was done with E-Prime 2 (PST, Pittsburgh, PA). Each participant was tested individually at 70cm distance from a 15-inch computer screen.

#### Attention bias assessment

Attention bias assessment using the dot-probe task was done at pre and post-treatment. The task consists of 120 trials (80 angry-neutral and 40 neutral-neutral). Each trial begins with a fixation cross (500 ms; white cross 1*1 cm at the centre of the screen), on which the participants are instructed to focus their gaze. Then, a face pair display of 500ms duration follows. Next, a small visual probe (< or >) appears at the place of one of the two faces. Participants must determine which symbol appeared by clicking right or left on the mouse, as quickly as possible. The target probe remains on the screen until there is a response, which starts a new trial, after an inter-trial interval of 500ms. Presentation of angry-face location, probe type, location and actor are fully counterbalanced. Less than 70% accuracy determines experiment abortion. In this case, a warning gives the opportunity for the experiment to start from the beginning. No participant had to start over in this study. Task completion takes about 5 minutes.

Although the optimal way to analyze dot-probe data continues to be discussed, in the current study, for purposes of comparability to previous ABM work on SAD, we relied on the most widely used method to assess attention biases, i.e. the bias score [e.g., 14,48,49], despite concerns expressed for its reliability [40,41]. The reaction time on stimuli which replace the threatening face when it is presented with a neutral (i.e., congruent trials) is subtracted from reaction time on stimuli which replace non-threat (neutral face) when it is presented with threat (angry face) (i.e., incongruent trials). A positive number reflects faster identification of threat. However, a negative number reflects quick detection of neutral stimuli and is interpreted as avoidance of threat [23,25,34,66].

#### ABM and placebo protocols

ABM and placebo protocols consist of 160 trials (120 angry-neutral and 40 neutral-neutral presentations) of a similar dot-probe task. In the placebo condition, angry-face location, probe location, and actor are fully counterbalanced in presentation. The only aspect that changes in the ABM protocol in relation to placebo is that in all angry-neutral pairs the probe is presented only after neutral faces, providing training away from threat. Additionally, probe type (< or >) is not factorially counterbalanced but there is equal probability of presentation for each of the following: angry-face location, probe location, or actor. Every 40 trials there is a short break. If accuracy is < 70%, a warning is presented during the break, giving an opportunity for the experimenter to remind the participant not to compromise accuracy. The protocol takes about 7 minutes for completion.

Reliability of trials in the current sample was good at pre-treatment (baseline assessment): neutral-neutral trials: Cronbach’s alpha = 0.94 and split-half reliability Spearman *r* = 0.90, angry-neutral congruent trials: Cronbach’s alpha = 0.94 and split-half reliability Spearman *r* = 0.85, angry-neutral incongruent trials: Cronbach’s alpha = 0.94 and split-half reliability Spearman *r* = 0.86 as well as at post-treatment: neutral-neutral trials Cronbach’s alpha = 0.83 and split-half reliability Spearman *r* = 0.77, angry-neutral congruent trials: Cronbach’s alpha = 0.81 and split-half reliability Spearman *r* = 0.75, angry-neutral incongruent trials: Cronbach’s alpha = 0.85 and split-half reliability Spearman *r* = 0.80. However, test re-test reliability was low for neutral-neutral trials *r* (60) = 0.40, *p* < 0.01, angry-neutral congruent trials *r* (60) = 0.33, *p* < 0.01, and angry-neutral incongruent trials *r* (60) = 0.37, *p* < 0.01.

Additionally, split half reliability of bias score was low r = -.22 at pre-treatment and post-treatment r = .08. Similarly, there was low test re-test reliability of bias score *r* (60) = -0.11, *p* = 0.70. The same levels of reliability have been found in studies [67,68] in undergraduate students and studies [27,69] in socially anxious individuals. There is an exception study [70] in a civilian population after war, which found high split-half reliability of bias score. Nonetheless, in an effort to be comparable with previous studies [23,25] with the same version of the dot-probe task, as well as all the studies assessing ABM effectiveness [exemption is 27], the traditional way of calculation was used for the current study.

### Data analysis

Initially, frequency analysis and one-way ANOVA was done to examine the attention bias levels of participants and differences in their social anxiety levels respectively. T-test on assessed characteristics was used to verify participants’ random assignment to group. To be consistent with other studies [e.g., 23] Generalized Estimating Equations (GEE) [71,72], was used in to examine ABM effects. This analysis accounts for correlated repeated measurements and accommodates missing data under the missing- at-random assumption. Specifically, it computes estimated marginal means, thus is considered as an intention-to-treat analysis including data from all randomized participants who gave at least one data point. To represent within-subject dependencies in the models, we specified an unstructured covariance matrix. Effect of changes on attention bias score and social anxiety levels were estimated using models containing main effects of group and time, and their interaction. The moderating role of initial attention bias level (continuous variable) was assessed using moderation analysis in the PROCESS macro of SPSS (SPSS Inc., Chicago, Illinois, USA), with Group (ABM, placebo) as predictor variable and difference score on social anxiety measures between pre- and post-treatment as outcome variables [as in 25]. Finally, based on [73] in order to achieve power of 0.80 with α = 0.05, two tailed, and obtain the smallest effect found in previous studies (*d* = 0.58) [26] a sample size of 26 participants was indicated to capture changes in pre to post-treatment effects.

## Results

### Descriptive analyses

Frequencies showed that most of the participants, N = 33, presented limited attention bias (i.e. attention bias score close to zero (M = 1.73, SD = 7.30). Fifteen of them presented mostly Avoidance (M = -26.21, SD = 15.82) and 18 of them presented mostly Vigilance (M = 28.54, SD = 14.55) (1/2 SD below or above the Mean). There was a statistically significant difference on SPAI-23 difference score between groups formed on the basis of their attention biases, one way-ANOVA, *F* (2, 58) = 3.26, *p*<0.05. Specifically, participants with attention bias scores close to zero presented the highest social anxiety levels (M = 34.22, SD = 8.71), followed from those with vigilance (M = 32.65, SD = 6.14) and those with avoidance (M = 27.73, SD = 8.28). Post-hoc tests showed that this difference was significant only between those with limited attention bias and avoidance, *p* < 0.05 and not between those with vigilance and avoidance *p* = 0.26. Differences on the LSAS total were not significant, *F* (2, 51) = 0.41, *p*<0.66.

### Intervention Group equivalence

Preliminary analyses indicated no Group (ABM, placebo) differences in baseline attention bias level t(58) = 1.33, p = 0.19, SPAI-23 difference score t(57) = -1.75, p = 0.09, or LSAS total t(50) = -0.80, p = 0.43 (see Table 1).

**Table 1:** Group equivalence data.

Group		
Variable	ABM (N = 32)	Placebo (N = 28)
Threat bias	-0.98 (23.45)	7.10 (23.60)
SPAI difference score	33.87 (7.72)	30.18 (8.52)
Liebowitz score	66.65 (26.22)	61.04 (24.43)

Note: Values in parentheses represent Standard Deviations. Threat bias = The reaction time on stimuli which replace the threatening face, when it is presented with non-threat (i.e., congruent trials), subtracted from reaction time to stimuli which replace non-threat (neutral) face when it is presented with threat (i.e., incongruent trials).

### Pre to post treatment effects

GEE showed no effect of Time, *Wald* χ^2^(1) = 0.02, *p* = 0.88 and no main effect of Group, *Wald* χ^2^(1) = 1.22, *p* = 0.27 on attentional biases, and no statistically significant interaction between Group and Time, *Wald* χ^2^(1) = 0.93, *p* = 0.34.

Results also showed no significant changes in social anxiety from pre to post treatment on any of the self-report measures: Specifically, there were no significant effects of Time, *Wald* χ^2^(1) = 0.49, *p* = 0.48, Group, *Wald* χ^2^(1) = 2.51, *p* = 0.11, or Group x Time interaction, *Wald* χ^2^(1) = 0.29, *p* = 0.59 on SPAI-23 difference scores. Similarly, there were no significant effects of Time, *Wald* χ^2^(1) = 1.81, *p* = 0.18, Group, *Wald* χ^2^(1) = 0.37, *p* = 0.54, or Group x Time, *Wald* χ^2^(1) = 0.96, *p* = 0.33 on LSAS total.

Finally, there was no significant change in ADIS-IV diagnostic status from pre to post treatment, as all participants maintained the same SAD status (general, performance).

### Moderating role of baseline attention biases

The moderation analysis using the command process.spd, with Group as the predictor variable (ABM, placebo), difference SPAI-23 score between pre-treatment and post-treatment as the outcome, and baseline attention bias level (continuous) as the moderator variable, showed that the model was not statistically significant F (3, 45) = 0.88, p = 0.46. Similarly, a non-statistically significant model was obtained when moderation was examined for LSAS difference score from pre- to post-treatment, as the outcome variable, *F*(3, 45) = 0.88, *p* = 0.46.

## Discussion

Previous studies show mixed results with regards to the effectiveness of ABM for social anxiety, an effect that needs further clarification, especially in light of evidence that not all socially anxious individuals show the expected levels of hyper-vigilance to threat, as demonstrated in previous studies [38,41], as well as by the present data. Although heterogeneity in the type of attention bias associated with SAD is still the subject of discussion, with some authors strongly suggesting that it may occur dynamically within individuals, over the duration of the task, rather than at an individual difference level [40,41], for the current study we focused on baseline attention bias as an individual difference between participants, in order to examine whether this moderates ABM effects, in an effort to be comparable with almost all the studies measuring ABM effectiveness [exemption is 27]. To the degree that individuals may differ in the degree to which they are characterized by vigilance vs avoidance, ABM may prove more helpful for some and not for other socially anxious individuals [33]. Consideration of the effects of baseline tendencies towards one rather than the other type of attention bias can lead to more effective protocols. Studying such effects in a controlled experimental setting, with individuals who are not treatment seekers can allow for the control of more factors than in a clinical trial, that may contribute towards identifying the mechanisms of effects.

We found that almost half of the participants presented with limited attention bias to threatening faces, with scores close to zero when taking their average bias score across trials, in comparison to the neutral stimuli; of the remaining participants almost half presented vigilance and half avoidance, indicating that it is only a subset who show clearly and uniquely bias towards threatening faces. As the participants with close to zero scores seemed to have the highest levels of social anxiety, it should be noted that they may have presented equivalent degrees of both vigilance and avoidance (i.e. as per dominant vigilance-avoidance models [48,49,74], which cancel each other out in the typical way attention biases are calculated. This tentative interpretation is consistent with previous arguments of dynamic, within individual variation in attention bias types, that may occur at the trial-by-trial level, an effect which is disguised when taking the average over large blocks of trials [40,41], and which requires serious consideration in future ABM work.

Notably, and in accord with a substantial subset of similar studies, ABM was not effective in changing threat bias and social anxiety levels of socially anxious college students. Specifically, no attentional biases (threat bias) changes were found from pre- to post-treatment, unlike several previous studies, and as a result there were no significant changes from pre- to post-treatment with regards to social anxiety status on the ADIS or self-reported anxiety. Against initial hypotheses baseline attention bias level did not moderate ABM effects. It is possible, that future work that takes into account the dynamic changes in attention biases within individuals can better clarify the patterns of bias on which ABM is most effective, and the relation of these patterns to experienced and reported social anxiety symptoms.

This study is inconsistent with limited evidence of effectiveness in a college sample [21]. However, the current study is not the only one finding no effectiveness of ABM for SAD (and anxiety in general), and negative findings like this may be important in delineating the circumstances under which the treatment is indeed effective. Furthermore, most studies showing treatment gains with regards to social anxiety levels, observed these on clinician administered measures [23,26] or state anxiety levels measured by behavioral assessment and self-report [19] and not on self-report measures of social anxiety, or clinical interviews at post-treatment. However, even when self-report measures of social anxiety showed changes from pre to post-treatment, social anxiety levels were still above the clinical cut-off at post-treatment [13]. Although more studies would be needed to make this claim, it is possible that removing the effect of treatment-seeking and the potential expectancy effect of believing that one receives an effective treatment, may be associated with null or reduced effectiveness. Our negative findings are in accordance with studies that found no differences between the ABM and placebo group on self-report, clinician interview and behavioral measures of social anxiety [30], and with studies that used a different delivery method, delivering ABM online, which may be less comparable to the present study [e.g. 28,29,31,75]. In all, this study builds on an accumulating body of evidence that ABM may be less effective for SAD than other types of anxiety, for which much stronger effects were obtained. However, results prompt the need for further research on potential moderators of effectiveness, including via a more specific delineation of attention bias heterogeneity.

With regards to effectiveness of ABM in changing attention biases from pre-treatment, out null results are more difficult to explain, given the positive results in many previous studies [76]. Although our study is not the only one reporting no attention bias changes from pre- to post-treatment [e.g. 23], the reasons for the negative effects deserve further examination. It has been argued that participants with limited attentional biases toward threat do not present with gains in comparison to the control group [50], suggesting that attention biases may not be the mechanism sustaining anxiety in this case, and the need for alternative treatment options. However, we had also observed that individuals showing limited attention bias at baseline, in fact, scored higher in social anxiety, which may indicate that they should be the ones demonstrating greater gains. As we hypothesized, heterogeneity of attention biases in social anxiety and the type of bias favored by each participant may have affected present results, although moderation was not supported. This may be because attention bias should be assessed using a dynamic approach at the trial level, an approach that would yield rich data to help identify who, and under what circumstances responds best to ABM [41].

Results of the present study should be seen in light of some limitations. First, with regards to examining the effects of baseline attention biases, a larger sample may have been necessary to detect significant interactions, therefore, the absence of effects here should be considered as preliminary. The sample was comprised of participants with general social fears and performance anxiety; the mixed nature of the group may have affected the effect of ABM, which may be more potent in the more severe form of social anxiety, the general subtype [2]. It should be noted that an exploratory examination of ABM effectiveness separately for the two groups, not reported here for brevity, showed no difference between them. Additionally, the pre-selection of participants may need to be considered crucial at the examination of AB at the individual difference level, so that larger and equal numbers of participants primarily showing vigilance vs avoidance take part, in order to ensure adequate power to detect moderation effects.

A more general issue pertains to the use of the dot-probe task, which, despite its widespread use, has been questioned in recent studies for its reliability [e.g. 40,41] as well as it has shown low reliability in the current study. Reliability issues may account for mixed findings regarding attentional bias and anxiety reduction, and the null finding for the current study. Adopting newer, and potentially more reliable ways of calculating attention biases on the dot-probe task [41,77], over the duration of the task rather than as an overall mean may be warranted. Therefore, it may be useful in future studies to take such a dynamic perspective in analysing dot-probe task data, to more accurately reflect heterogeneity in attention biases in social anxiety, and its role in predicting ABM effectiveness. Nonetheless, the reliability of dot-probe task needs more investigation with regards to the length of it and how this might affect its reliability [78] e.g. the dynamic approach of analysing dot-probe task creates a higher number of trials which in turn might be related with the good reliability. In defence of our reliance on the more traditional approach to analysis in the current study, we aimed for comparability to the previous work on SAD, that yielded mixed effects of ABM, in order to address the role of variables like individual differences, treatment seeking and types of outcome measures. Adding to that, relying on methods beyond reaction time, such as eye-tracking may be another way to circumvent such concerns. A final point with regards to limitations, is that several participants reported fatigue by the end of the task, which may have altered their attention allocation. Generally, participants’ experience with the dot-probe task is an issue that limited studies examined [79], a topic that may prove useful to consider in future work [80].

## Conclusions

In sum, this experimental study of the effects of ABM in social anxiety found no significant improvement in either attention biases or in self-report and clinical interview measures of social anxiety. Additionally, baseline attention biases did not moderate social anxiety changes from pre- to post treatment, even though they strongly suggested the presence of individual differences in whether individuals show mostly a vigilance or an avoidance pattern. The heterogeneity of attention biases, together with methodological differences from previous studies that found effectiveness of ABM in SAD should be taken into consideration in future research to produce more conclusive results as to if, under what circumstances, and for whom, this form of training is an effective intervention.
